# A machine learning model to predict privacy fatigued users from social media personalized advertisements

**DOI:** 10.1038/s41598-024-54078-w

**Published:** 2024-02-14

**Authors:** Ghadeer Alwafi, Bahjat Fakieh

**Affiliations:** Information Systems Department, King Abdulaziz University, Jeddah, 21589 Saudi Arabia

**Keywords:** Computational science, Computer science, Scientific data, Statistics, Psychology, Human behaviour

## Abstract

The increasing use of social media platforms as personalized advertising channels is a double-edged sword. A high level of personalization on these platforms increases users’ sense of losing control over personal data: This could trigger the privacy fatigue phenomenon manifested in emotional exhaustion and cynicism toward privacy, which leads to a lack of privacy-protective behavior. Machine learning has shown its effectiveness in the early prediction of people’s psychological state to avoid such consequences. Therefore, this study aims to classify users with low and medium-to-high levels of privacy fatigue, based on their information privacy awareness and big-five personality traits. A dataset was collected from 538 participants via an online questionnaire. The prediction models were built using the Support Vector Machine, Naïve Bayes, K-Nearest Neighbors, Decision Tree, and Random Forest classifiers, based on the literature. The results showed that awareness and conscientiousness trait have a significant relationship with privacy fatigue. Support Vector Machine and Naïve Bayes classifiers outperformed the other classifiers by attaining a classification accuracy of 78%, F1 of 87%, recall of 100% and 98%, and precision of 78% and 79% respectively, using five-fold cross-validation.

## Introduction

Social media play a significant role in people’s lives for several purposes, such as communication, education, employment, and entertainment. A broad global survey conducted in 2023 of internet users showed that the number of active social media users reached 4.8 billion, which is equivalent to more than half of the world’s total population^1^. People of different ages, occupations, education levels, backgrounds, geographical locations, and cultures use these platforms, providing a valuable opportunity for businesses.

The fact that personalized ads on social media are beneficial for the customer is not ignored; 80% of customers reported that when marketers personalized the experience, they were more likely to purchase^2^. However, social media ads lead to privacy issues, including privacy fatigue. Privacy fatigue is a relatively novel psychological phenomenon that refers to users’ boredom with online privacy issues and requirements, which leads to a lack of privacy-protective behavior^3–5^. The dimensions of privacy fatigue are cynicism and emotional exhaustion^5^. Studies showed that privacy fatigue affects users’ behavior more than privacy concerns^5,6^.

Several factors could affect privacy fatigue, such as users’ Information Privacy Awareness (IPA) and personality traits^6–8^. In this study, IPA refers to users’ awareness of the personal data that is collected, used, and shared for social media ads and the impact of this practice in the future. According to personality psychologists, there are five basic dimensions of personality, known as “the big five”: extraversion, neuroticism, openness, conscientiousness, and agreeableness^9^.

The use of Machine Learning (ML) and social media data to predict human behavior and emotions, such as cyberbullying, anxiety, depression, and concerns is achieving tremendous attention among researchers^10–13^. It is mainly used to prevent, avoid, or reduce the impact of these issues. Previous research has focused on privacy concerns regarding social media ads^13–16^. Therefore, with only a few studies on the phenomenon of privacy fatigue^5,6,17^, this study extends the concept into the social media context. It aims to predict users who will be affected by high privacy fatigue, based on their IPA and personality traits, so that they can avoid the lack of privacy-protective behavior and the consequences of failing to take appropriate precautions.

Therefore, the objectives of this study are as follows: (a) find whether the privacy fatigue psychological state exists regarding social media ads; (b) examine the relationship between privacy fatigue and users’ IPA level regarding the personal data collected, used, and shared for social media ads; (c) discover whether users’ personality traits have an impact on privacy fatigue; and (d) develop a model using a machine learning classification algorithm to predict users with privacy fatigue due to social media ads, based on their IPA and personality traits.

## Related works

### Privacy fatigue

With the evolution of modern communication technologies, information privacy has been under increasing threat and has become an area of interest for researchers. A relatively novel phenomenon regarding online privacy called privacy fatigue is currently under discussion by researchers^5,6,17^. This type of fatigue is psychological. The concept of psychological fatigue was introduced first in the medical field to reflect unpleasant feelings and tiredness resulting from high expectations and unmet demands^18,19^. Psychological fatigue has been explored in several research fields, such as clinical research^20^, occupational research^21^, and recently online research due to technological advances^5–7^.

Online privacy fatigue is a psychological state that reflects an individual’s sense of weariness toward online privacy issues; they believe that there is no effective means of managing their personal information and maintaining privacy on the Internet^3–5^. The two key dimensions of online privacy fatigue are cynicism and emotional exhaustion^5^. Cynicism is defined as “an attitude toward an object accompanied by frustration, hopelessness, and disillusionment”^5^. Emotional exhaustion indicates a “chronic state of emotional and physical depletion”^5^.

Privacy fatigue has various causes and effects. The causes of this state are the complexity of measures to manage individuals’ own personal data, feeling a loss of control over these data, and frequent exposure to data breaches^5^. The effect is that users with privacy fatigue tend not to engage in privacy-protective behavior^17^.

Few studies, as illustrated in Table 1, have explored privacy fatigue, its antecedents and/or consequences, despite its strong effect on behavior. Most of the existing privacy fatigue studies discuss contexts that contain massive amounts of sensitive personal information, such as mobile apps^6^, mHealth^22^, e-government^17^, and the Internet of Things (IoT)^8^. Additionally, several antecedents that induce privacy fatigue have been proposed, such as personality traits^6^, privacy policy effectiveness^22^, users’ knowledge^8^, and privacy information transparency^17^.

**Table 1:** Privacy fatigue studies.

The study	Purpose	Findings	Context
^6^	Explore the effect of privacy fatigue, privacy concerns, and personality traits on users’ intention to disclose personal information	Some personality traits have an effect on privacy concerns and privacy fatigue	Mobile apps
		Privacy fatigue has a greater impact on intention to disclose personal information than on privacy concerns	
^22^	Explore the effect of privacy paradox from the perspective of privacy calculus and privacy fatigue on disclosure intention	Perceived benefits have a greater impact on disclosure intention than privacy concerns	mHealth apps
		Privacy fatigue has an insignificant impact on disclosure intention	
^8^	Analyze privacy fatigue phenomenon in an IoT environment	Low IoT security knowledge level is likely to increase privacy fatigue	IoT
		Privacy fatigue could feel different, depending on the IoT devices’ usage and purpose	
		Frequent failure to protect privacy can increase privacy fatigue	
^17^	Examine the effect of privacy information transparency to mitigate privacy fatigue	Privacy information transparency positively impacts both dimensions of privacy fatigue, i.e., cynicism and emotional exhaustion	E-government

### Prediction of psychology using ML

The prediction of human behavior, personality, and emotions in general, and using data from social media platforms, in particular, is receiving tremendous attention among researchers^23^. Some of these studies are summarized in Table 2. For instance, ML is used to predict aggressive behaviors on these platforms, such as cyberbullying. A review of cyberbullying prediction studies found that the main data collection strategy used data extracted from social media, either by using keywords, such as hashtags, or by using user profiles^10^. The study also stated that the ML algorithm most often applied in cyberbullying prediction is Support Vector Machine (SVM), followed by Naïve Bayes (NB).

**Table 2:** ML algorithms used for behavior and psychology prediction based on social media data.

The study	Purpose	Algorithms used
^10^	A review study of ML algorithms used for cyberbullying prediction on social media	Most used algorithm is SVM followed by NB
^23^	Predict personality traits based on social media status	Maximum Entropy
^9^	Predict personality traits based on social media photos	RF, ET, and GBT
^11^	Predict anxiety level during COVID-19	SVM and DT
^12^	Predict depression based on Facebook data	SVM, DT, and KNN

Social media users’ posts, comments, and likes are used to study and predict users’ personality traits. One study predicted personality traits based on color preference and posted photos and found a relationship between them^9^. The study used tree-based algorithms, such as Random Forest (RF), for the proposed model. The result showed that all the models performed similarly and accurately, with no large errors or outliers in the prediction. This study used personality traits as response variables, whereas other studies used them as explanatory variables. For example, a study^24^ considered users’ personality traits and smartphone usage for happiness recognition.

ML is also used to predict human emotions, such as anxiety and depression. A study was conducted in Saudi Arabia to predict anxiety levels during the COVID-19 pandemic for the purpose of preventing potential public mental health crises^11^. Data were collected using an online questionnaire and the model was trained by using these data, employing SVM and Decision Tree (DT) algorithms. The results showed that the proposed ML models demonstrated promising potential for early prediction of anxiety. Additionally, a paper^25^ reviewed 20 studies that used Artificial Intelligence (AI) and ML techniques to detect, predict, and analyze depression from social media platforms to prevent consequences such as suicide.

One of the reviewed studies developed a prediction model for depression using several classifiers, such as SVM, DT, and K-Nearest Neighbors (KNN)^12^. The outcome showed that SVM performed better compared to other classifiers, whereas regarding precision of features, recall, and F-measure, DT performed better.

### The current gap

Privacy issues regarding social media ads seem attractive to scientific researchers. The previous studies in this field mainly focused on privacy concerns^13,15,16,26^, privacy calculus^14,27^, and the personalization-privacy paradox^28,29^. Researchers stated that users’ negative emotions affect their privacy decisions and behaviors^6^, including both privacy concerns and fatigue. However, studies showed that privacy fatigue affects privacy behavior substantially more than privacy concerns^5,6^.

Privacy fatigue has spread widely; however, there are still few works existing on this phenomenon (Table 1)^5,6,17^. Thus, this research contributes to discovering the privacy fatigue phenomenon in the social media context, a gap that has not yet been explored. This is done by investigating whether the frequent collection and use of personal data for social media ads influences users’ privacy fatigue.

This study also contributes to the field of knowledge by examining whether privacy fatigue will continue, even if users have a high IPA level and if personality traits have an impact. According to the literature, an additional gap was found where these two privacy fatigue antecedents were discussed by only a few studies and in different contexts, which can be illustrated by two main points. First, the effect of users’ privacy awareness and knowledge of privacy fatigue was examined only in the context of the IoT^8^. Second, although personality traits were explored in both privacy fatigue and social media fatigue studies^7^, the privacy fatigue study examined personality traits only in the context of mobile apps in general^6^.

Additionally, to the reader’s knowledge, ML has been used to predict several human psychological issues, as discussed in “Prediction of psychology using ML" section, but not privacy fatigue, which highlights the last gap. Therefore, this research contributes to predicting users with privacy fatigue using ML, where users’ IPA level and personality traits play a significant role as predictors.

## Research model and hypothesis development

**Figure 1:**
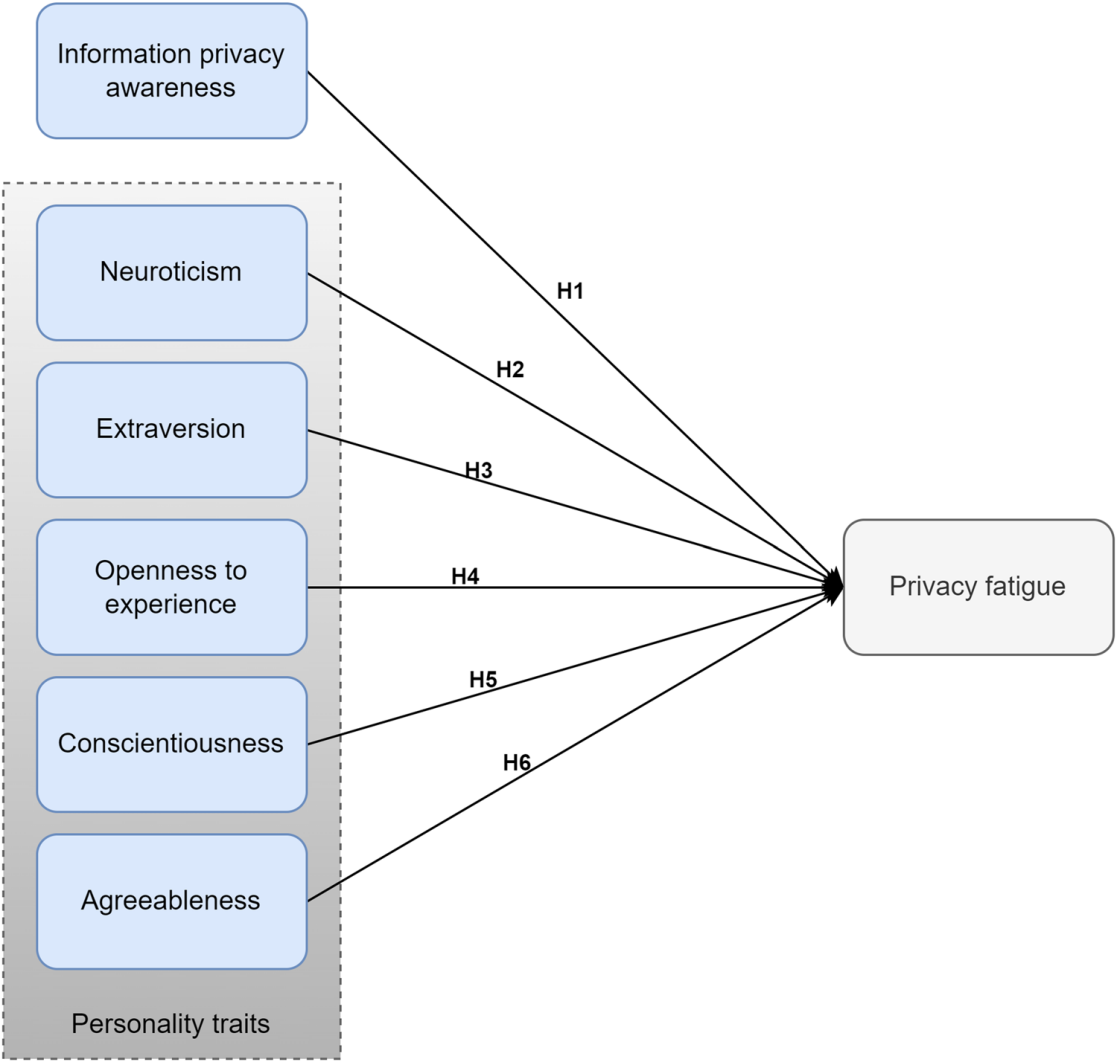
The theoretical model.

The model of this study is shown in Fig. 1. Details regarding the study hypothesis are illustrated next.

### Information privacy awareness

Users with privacy fatigue suffer from a lack of privacy-protective behavior^5^. A previous study^17^ explored the literature to find factors that motivate such behavior to examine its effect on privacy fatigue and found that information transparency motivates privacy behavior. The study’s results confirmed lower privacy information transparency would increase cynicism and emotional exhaustion. From the literature, IPA also motivates privacy-protective behavior^30^, which is needed for users who suffer from privacy fatigue. A study that explored the privacy fatigue phenomenon in the IoT environment found that users’ security awareness and knowledge about IoT affected their privacy fatigue^8^, such that a user’s low level of IoT security knowledge is likely to increase privacy fatigue. Based on that, the first hypothesis of this study is:

#### H1

IPA level has a significant relationship with privacy fatigue.

### Personality trait

Personality traits have been associated with privacy fatigue. A study of privacy fatigue antecedents and consequences suggested that future research must incorporate personality traits to understand better which social personality traits of media users make them highly susceptible to experiencing fatigue^31^. A study^7^ examined only the moderating role of neuroticism and extraversion traits. They found from the literature that these two traits may influence social media fatigue the most^7^. Their results showed that extroverted users are less likely to be concerned regarding privacy. On the other hand, neurotic users feel more insecure and disturbed and have intense feelings of invasion of privacy. However, they reported the limitation of examining only two traits.

Additionally, a study^6^ found that all five personality traits were significantly related to privacy fatigue, with neuroticism being positively related, and the other traits negatively related. Therefore, it is hypothesized that:

#### H2

Neuroticism has a significant relationship with privacy fatigue.

#### H3

Extraversion has a significant relationship with privacy fatigue.

#### H4

Openness to experience has a significant relationship with privacy fatigue.

#### H5

Conscientiousness has a significant relationship with privacy fatigue.

#### H6

Agreeableness has a significant relationship with privacy fatigue.

## Materials and methods

### Procedure

**Figure 2:**
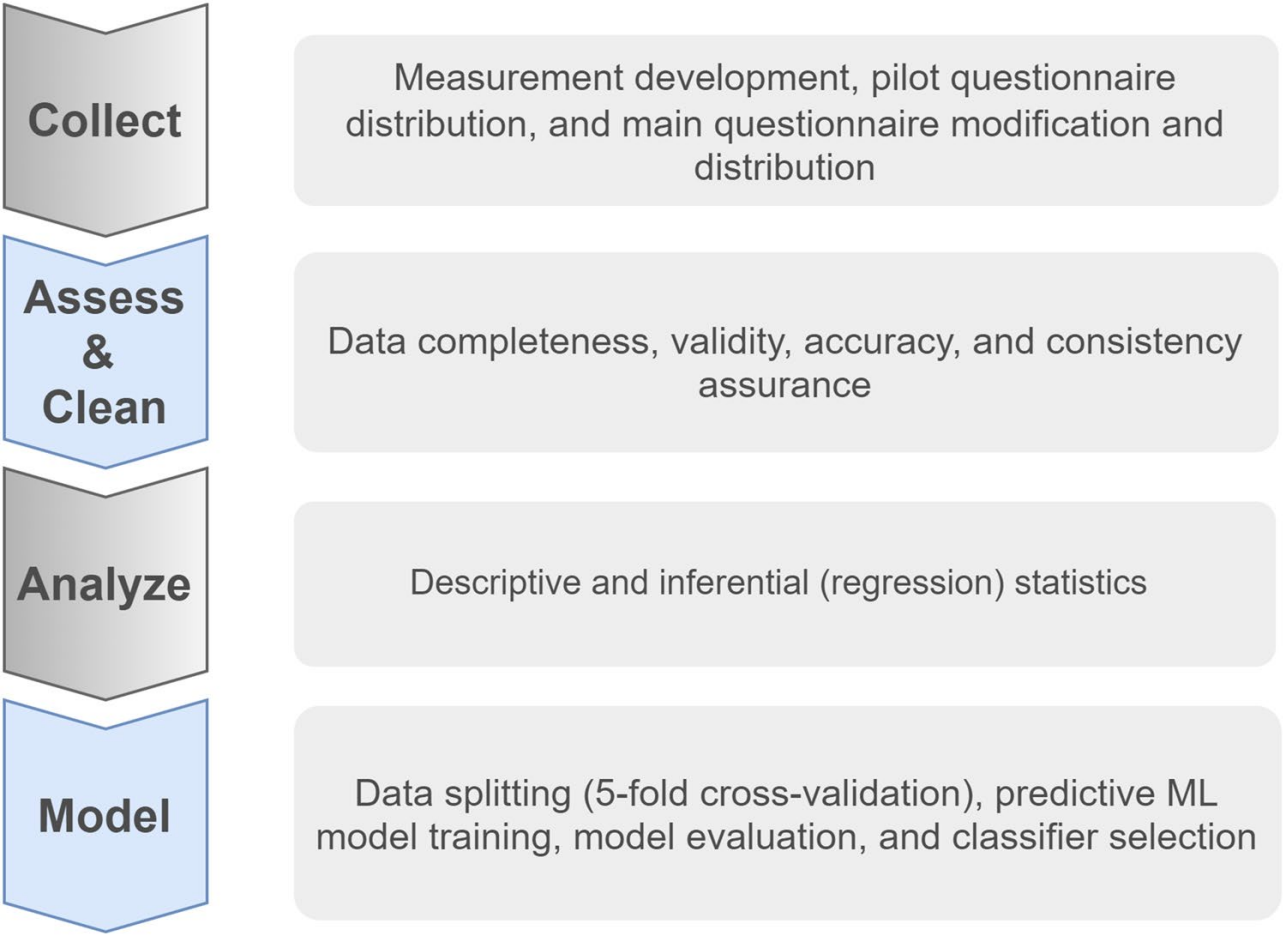
Methodology brief.

The four main stages followed by this study are Collect, Assess and Clean, Analyze, and Model (Fig. 2). The Collect stage starts by finding the appropriate instrument to collect the required data. The selected instrument was a questionnaire adopted from the literature. Minor modifications were made to the adopted measurements to match the study purpose, along with English-to-Arabic translation. The evaluation method of back-translation was used to ensure translation quality^5,7^. Before the main questionnaire distribution, a pilot questionnaire was carried out and final modifications were made to address challenges and ensure clarity and reasonable response time. It was conducted using a convenience sampling method. The data collected from the pilot study was not included in the main study to avoid bias issues associated with convenience sampling^32^. Thereafter, the main questionnaire was distributed.

The Assess and Clean stage started after receiving a number of responses and closing the questionnaire. The main purpose of this stage is to facilitate and improve the Analyze stage to get valid and reliable results. Therefore, data assessment was carried out to address issues related to data content and structure, i.e., completeness, validity, accuracy, and consistency. In the Clean stage, the following has been done:

- Delete unnecessary columns.
- Rename columns to short, meaningful names.
- Delete some records based on exclusion criteria (e.g., untargeted users and identical responses).
- Remove Arabic text.
- Transform answers to a numerical scale.
- Fill in missing values with the most frequent answer.
- Correct some "Other" answers if the answer is already in the options above.
- Reverse the scale of some personality trait questions.
- Split some columns.

The Analyze stage included descriptive statistics and inferential statistics. Descriptive statistics include finding Mean, Median, Standard Deviation (SD), maximum (Max), and Minimum (Min), while inferential statistics include discovering *p*-values and coefficients. These statistics helped to find the relationships between variables and validate the hypothesis.

The previous stages built a solid basis for the Model stage, in which an intelligent ML model was built to predict users who were likely to suffer from privacy fatigue. This involved splitting the data using five-fold cross-validation, training the model using several classification algorithms based on the literature, i.e., SVM, NB, KNN, DT, and RF, evaluating the classifier accuracy, recall, precision, and F1, and finally selecting the most accurate algorithm based on the evaluation results.

### Measurement development

Measurements of all the study variables were derived from related works^5,33–35^. Some of the questions were slightly modified to match the study’s purpose and scope (Table 3). All questions were measured using a five-point Likert scale ranging from “Strongly disagree” to “Strongly agree”.

**Table 3:** Measurement of the variables.

Variables	Items	Sources
Privacy fatigue	I feel irritable when dealing with privacy issues associated with social media ads (e.g., using my personal information on these ads)	^5^
	I think I am tired of privacy issues regarding social media ads	
	It is tiresome for me to care about privacy on social media	
	I have become less interested in privacy issues regarding social media ads	
	I have become less enthusiastic about protecting personal information provided to social media	
	I doubt the significance of privacy issues on social media more often	
IPA	I am aware that social media platforms are collecting too much personal information about me	^33,34^
	I am aware that when I give personal information to social media platforms for some reason, it would be used for other reasons such as ads	
	I am aware that social media platforms would sell and share my personal information with advertising companies	
	I am aware of how my personal information on social media platforms will be collected, processed, and used for advertising purposes	
	In general, I’m aware that it would be risky to use my personal information for social media ads	
	I am aware that there would be a high potential for loss associated with the collected, shared, and used personal information by social media for advertising purposes	
	I am aware that collecting, sharing, and using my personal information for social media ads would lead to many unexpected problems	
	I am aware that there would be too much uncertainty associated with the collected, shared, and used personal information by social media for advertising purposes	
Personality trait	I see myself as someone who is outgoing, sociable	^35^
	I see myself as someone who is generally trusted	
	I see myself as someone who is relaxed, handles stress well	
	I see myself as someone who is dependable, does a thorough job	
	I see myself as someone who has few artistic\creative interests	
	I see myself as someone who has an active imagination	
	I see myself as someone who is reserved, withdrawn	
	I see myself as someone who tends to be lazy	
	I see myself as someone who gets nervous easily	
	I see myself as someone who is critical, tends to find fault with others	

Fatigue, in general, is characterized by emotional exhaustion and cynicism, as discussed in the literature^5^. Therefore, the adopted scale measuring individuals’ privacy fatigue is based on these two core dimensions, each of which has 3 items to measure it^5^. This scale was selected because it was the first scale developed to measure privacy fatigue; moreover, it has been adopted by all the subsequent studies and its validity and reliability have been reported^17,22^.

To measure IPA, two models were combined. Several studies have measured IPA by measuring privacy concerns, assuming that individuals’ concerns about future risks could be associated with their awareness of potential risks^36,37^. However, to measure IPA directly, a model called Information Privacy Awareness (IPA) indicated that researchers should consider three aspects that make up privacy awareness^34^. These aspects cover the awareness of:

1. The element related to information privacy.
2. The element’s existence in the current environment.
3. The element’s impact, where the element could be technology, regulation, or practice.

In this study, the element is practice: The users’ awareness level of the practice of collecting, using, and sharing personal data for social media ads was measured.

Considering these three aspects, a scale called the Information Privacy Concerns (IPC) model, was adopted^33^. The model is comprehensive and has been employed by several studies to measure internet privacy concerns, where awareness researchers chose the appropriate dimensions and items of the model based on the purpose of their research^22,30^. The model includes several dimensions of internet privacy concerns, such as personal information collection, unauthorized secondary usage, and improper access. Each dimension includes a set of valid and reliable items. Therefore, appropriate items were chosen from the IPC model^33^, with respect to the IPA aspects noted above^34^. Items were modified to address awareness instead of concerns, so as to measure awareness directly, as suggested by Correia and Compeau (2017).

To measure participants’ personality traits, the Big Five Inventory (BFI-10) scale was employed^35^. Considering participants’ limited time, there are two scales that offer only 10 items of the BFI: the Ten-Item Personality Inventory (TIPI) and BFI-10^35,38^. Both measure extraversion, neuroticism, openness, conscientiousness, and agreeableness traits using two items for each. Both have proved their simplicity, reliability, and validity. However, BFI-10 is the most recent and was clearer when translated into Arabic. Additionally, a study mentioned that the BFI-10 could be an option if the author is not interested in inferring individual differences^39^. This study does not aim to investigate individual differences but to capture the overall effect of personality traits on privacy fatigue.

### Sample

The demographics of the respondents are summarized in Table 4. The sampling technique used for the main study is probabilistic sampling, particularly simple random sampling^32^. This technique was used to ensure an unbiased random selection and a representative sample where each member of the population has an equal chance of being selected, which results in more accurate and generalizable results^40^.

**Table 4:** Demographics of the sample.

Measure	Items	Frequency	Percentage (%)
Gender	Female	399	79
	Male	109	21
Age	19 and below	42	8
	20–34	260	51
	35–44	111	22
	45 and above	95	19
Educational qualification	High school or below	89	17
	Diploma	40	8
	Bachelor’s degree	333	66
	Master’s degree or higher	46	9
Occupation	Student	116	23
	Employed at public/government organization	108	21
	Employed at private sector	116	23
	Self-employed	17	3
	Unemployed	112	22
	Retired	39	8
Social media usage frequency	Daily	502	99
	Weekly	4	1
	Monthly	0	0
	Less often	2	0
If daily, the average time spent on social media	1 h and a half or less	62	12
	2 h to 2 h and a half	113	23
	3 h or more	225	45
	I don't know	101	20

All methods were carried out in accordance with relevant guidelines and regulations as this research includes human participants. The experimental protocols and procedures were approved by the research committee of the Information Systems department at King Abdulaziz University, as it follows common and predefined regulations, and does not expose any personal information nor include any dangerous or harmful activities. However, the following guidelines were applied based on the committee’s recommendations to maintain confidentiality and anonymity, increase the response rate, ensure the quality of the data, and gain the respondents’ trust:

- The purpose of the study was declared at the beginning of the questionnaire.
- It was clearly stated that the data will be collected and used for study purposes only.
- It was made clear that respondents had the right to skip any question or to stop at any stage if they did not want to continue.
- Participation consent was required: therefore, respondents had the opportunity to agree or decline to participate.
- Questions that identified respondents were not included.

The main questionnaire was distributed from October 10, 2022, to October 30, 2022, through social media platforms such as Instagram, Snapchat, WhatsApp, and Telegram. The connections on the authors’ social media are mainly from the Arabian Gulf which indicates a specific targeted culture sample. The questionnaire applies to individuals proficient in Arabic and English as it was distributed in both languages. After completing the data cleaning, the number of responses obtained was 508 out of 538.

Female respondents formed the majority of the sample, at 399 individuals (79%). 260 participants fell into the 20–34 age group (51%), and 333 participants had a Bachelor’s degree (66%). The occupations showed close percentages, with 116 students (23%), 116 private sector employees (23%), 112 unemployed participants (22%), and 108 employees of public/government organizations (21%). Regarding the sample’s social media usage, 502 participants (99%) used social media daily and 225 respondents (45%) reported spending 3 h more on average on social media daily.

### Data analysis justification

The variables of this study, i.e., privacy fatigue, IPA, and personality traits are latent variables^41,42^. This implies that each variable is measured using multiple questions. Various methods can be used to analyze latent variables, such as structural equation modeling (SEM) or taking the sum/mean of all the questions’ scores for a particular variable^41,42^. The advantage of SEM is that it considers the reliability and validity of the questions during the analysis. However, with a small sample size, SEM will do poorly in discovering the actual effect^42^. Therefore, considering the sample size of this study and assuming that all questions are reliable and valid based on the literature, the mean of the answers of each variable was taken. There are 2 questions for each personality trait, 8 for IPA and 6 for privacy fatigue. The answers to these questions ranged from 1 to 5, as the options were a 5-point Likert scale. The average of each variable’s answers was calculated and used for analysis.

However, a classification for privacy fatigue was required in this study (0 or 1). Therefore, after taking the average, people who scored 3 or more were classified as having a medium-to-high level of privacy fatigue and labeled 1. Those scoring lower than 3 were classified as having a low level of privacy fatigue and labeled 0.

### Model development

Supervised machine learning was used to develop the prediction model, as it used a labeled dataset. Classification algorithms were applied to classify the users into two classes, i.e., low, or medium-to-high level, of privacy fatigue. The model predicts privacy fatigue based on 5 personality traits and IPA all holding values of 0 or 1. The dataset size is 508. As the dataset cleaning was conducted before the analysis, no further data preprocessing was required during the training.

For model development, several steps were taken. The classification algorithms that were used in this study are SVM, NB, KNN, DT, and RF. The selection is based on the algorithms used in the literature (Table 2). Other algorithms on the table were not selected: Maximum Entropy, GBT, and ET, because Maximum Entropy is a text classifier, which is not needed, while GBT and ET perform similarly to RF which is used in this study^9^.

K-fold cross-validation is considered the most reliable and profound method that helps avoid overfitted models, one of the most common issues in ML^43^. To get the best learning result and high accuracy, it is necessary to maximize the size of the testing and training dataset, which is what cross-validation does because it uses all the data for training as well as for testing^43^. In short, to perform cross-validation, the data is divided into a number of equal-sized subsets called folds. This is done in iterations, where each fold is removed once, and the rest of the folds are used as a training set. Then, the removed fold is used as a test. The accuracy of each iteration allows them to be compared, with highly divergent accuracy values indicating errors. The overall accuracy is calculated by taking the average accuracy of all the iterations. The average of F1, recall, and precision is also considered. Cross-validation using five or ten-folds is preferred based on empirical evidence^44^. In the previously discussed studies, three used ten-fold cross-validation^9,11,12^. However, five-fold cross-validation is used in this study as the dataset is not large.

### Ethical approval

The Information Systems Department research committee at King Abdulaziz University approved the experimental protocols and procedures. There were several measures advised by the committee presented in "Materials and methods" section. A written informed consent was obtained from the participants for the study before they conducted it. All methods were carried out in accordance with relevant guidelines and regulations.

## Results

### Descriptive statistics

The descriptive statistics of the study variables are summarized in Table 5. Personality traits and IPA values ranged from 1 to 5. The min value for most of these variables is 1, except for agreeableness and conscientiousness. The conscientiousness level started with 1.5, implying that no one in the sample considers themselves completely impulsive, careless, and disorganized. The min agreeableness score is 2; thus, the sample does not include people who 100% see themselves as critical, uncooperative, and suspicious. For all the variables discussed, the max value recorded is 5. Privacy fatigue, however, has only two values: the min value is 0, meaning that there are participants who have a low level of privacy fatigue, and the max value is 1, where participants have a moderate-to-high level of privacy fatigue.

**Table 5:** Descriptive statistics.

	Mean	Median	Mode	Standard deviation	Sample variance	Range	Minimum	Maximum	Count
Extraversion	3.4390	3.5	4	0.8561	0.7330	4	1	5	508
Agreeableness	4.0000	4	4	0.6374	0.4063	3	2	5	508
Neuroticism	2.6929	2.75	3	0.7865	0.6185	4	1	5	508
Conscientiousness	3.8051	4	4	0.7205	0.5191	3.5	1.5	5	508
Openness	2.8967	3	3	0.7986	0.6377	4	1	5	508
IPA	3.8216	3.875	4	0.6934	0.4808	4	1	5	508
Privacy Fatigue	0.7776	1	1	0.4163	0.1733	1	0	1	508

The mean of the IPA and personality trait variables ranged from 2.6929 to 4. The mean values for neuroticism and openness traits were 2.6929 and 2.8967, implying that the participants’ tendency toward unstable emotions and active imagination is moderate on average. Also, on average, participants have neutral to high levels of extraversion, conscientiousness, and IPA, with mean values of 3.4390, 3.8051, and 3.8216, respectively. Participants with the agreeableness trait are helpful, trusting, and empathetic, and these form the majority of the sample with an average value of 4. Additionally, on average, participants tend to have a medium to high level of privacy fatigue, with a mean value equal to 0.7776.

### Inferential statistics

For inferential statistics, regression analysis was conducted. This analysis is used to see how well the measures of personality traits and IPA level in the sample can predict the measure of privacy fatigue. The analysis is done by assessing several values, which are explained next.

The values that were explored are the coefficient of determination (R^2^), path coefficient, and path significance (*p*-value). R^2^ reflects the effect of all the independent variables combined on the dependent variable. Another way to view R^2^ is that it is a measure of the model’s predictive accuracy. This effect ranges from 0 to 1 where 0.75, 0.50, and 0.25 represent essential, moderate, and weak levels of predictive accuracy, respectively^45^. Path coefficients represent the relationships by values ranging from − 1 to + 1. Coefficients closer to + 1 indicate strong positive relationships, and coefficients closer to − 1 indicate strong negative relationships^45^.

In order to accept or reject the null hypothesis, the level of significance should be defined. In related studies, three significance levels are used, i.e., 0.001, 0.01, and 0.05^5,6,22^. Therefore, these three levels are also used in this study. *P*-values smaller or equal to these significance levels indicate a significant relationship, meaning that the null hypothesis is rejected.

**Figure 3:**
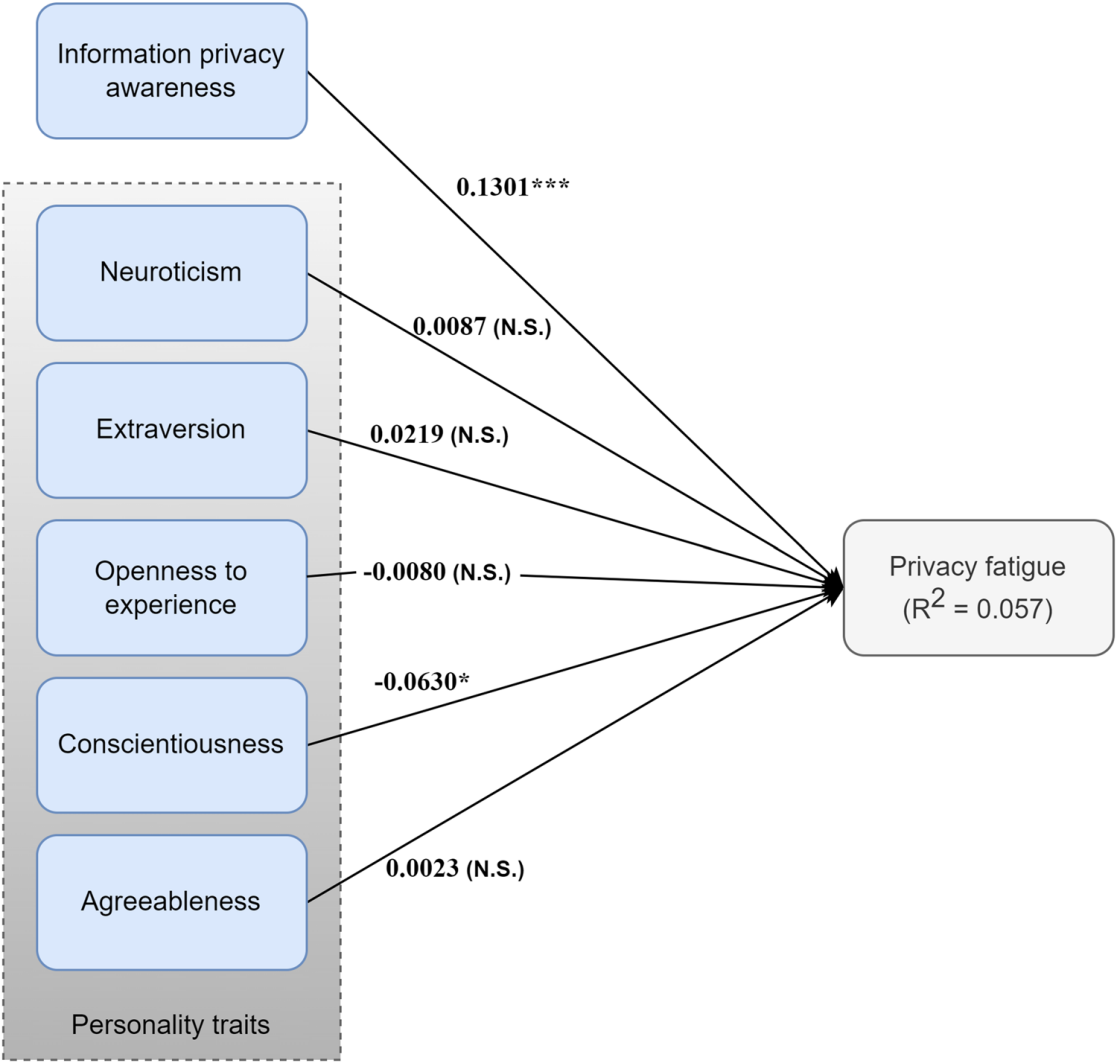
Theoretical model results (*p < 0.05,**p < 0.01,***p < 0.001, N.S., Not Significant).

As shown in Fig. 3, 2 relationships were significant, while 4 were not. The values on the arrows represent the path coefficient while the *p*-value is represented by one, two or three asterisks depending on the significance level, or by N.S if not significant.

The R^2^ result showed that around 5% of the variance in privacy fatigue was explained by IPA and personality traits, indicating a weak level of predictive accuracy.

IPA level was found to have a significant positive relationship with privacy fatigue (coefficient = 0.1301, *p* = 1.44*10^−6^). This indicates that an increase in IPA level results in an increase in privacy fatigue level.

Four personality traits out of five did not significantly affect privacy fatigue. Agreeableness had the most insignificant effect among other traits, with a coefficient equal to 0.0023 and a *p*-value equal to 0.9422. This was followed by neuroticism and openness, with similar *p*-values equal to 0.7327 and 0.7243, and coefficients in opposite directions equal to 0.0087 and − 0.0080 respectively. The last trait that had an insignificant effect on privacy fatigue was extraversion (coefficient = 0.0219, *p*-value = 0.3208).

Conscientiousness is the only personality trait found to have a significant negative effect on privacy fatigue (coefficient = − 0.0630, *p*-value = 0.0272). This means that an increase in the conscientiousness trait, i.e., people who are competent and dependable, resulted in a decrease in the level of privacy fatigue.

In summary, the null hypotheses of H1 and H5 are rejected. For H2, H3, H4, and H6, the findings failed to reject the null hypothesis. All results are summarized in Table 6.

**Table 6:** Hypothesis testing results (*p < 0.05,**p < 0.01,***p < 0.001).

Hypothesis	Path	Coefficient	Standard error	*P*-value	Decision
H1	IPA→privacy fatigue	0.1301	0.0267	1.44*10^−6^***	Reject the null hypothesis
H2	Neuroticism→privacy fatigue	0.0087	0.0255	0.7327	Fail to reject the null hypothesis
H3	Extraversion→privacy fatigue	0.0219	0.0220	0.3208	Fail to reject the null hypothesis
H4	Openness to experience→privacy fatigue	− 0.0080	0.0228	0.7243	Fail to reject the null hypothesis
H5	Conscientiousness→privacy fatigue	− 0.0630	0.0285	0.0272*	Reject the null hypothesis
H6	Agreeableness→privacy fatigue	0.0023	0.0316	0.9422	Fail to reject the null hypothesis

### Comparison of the models

The accuracy, F1 Score, recall, precision metrics were calculated to compare the models (Table 7). The SVM and NB models had the highest accuracy (78%), followed by RF (75%) and KNN (73%). The lowest accuracy was for the DT model, with 65%. These results imply that SVM and NB had the highest proportion of correct predictions out of the total number of predictions. Likewise, based on the F1 score, SVM and NB had the best F1 score (87%), followed by RF (85%), KNN (84%), and DT (77%), which means that SVM and NB models had better precision and recall compared to other models.

**Table 7:** Comparison of models.

Model	Accuracy	F1 score	Recall	Precision
SVM	0.78	0.87	1.00	0.78
KNN	0.73	0.84	0.91	0.78
DT	0.65	0.77	0.76	0.78
RF	0.75	0.85	0.93	0.78
NB	0.78	0.87	0.98	0.79

The recall percentage of the algorithms ranged from 76 to 100%. The SVM algorithm had the highest recall percentage, which tells us that 100% of the actual users with medium-to-high privacy fatigue were correctly identified. This result could be because of the robustness of SVM to the noise in the data, as the decision boundary (called hyperplane) is determined by the closest data points to the boundary (called support vectors)^46^. NB, RF, and KNN also had high recall percentages of 98%, 93%, and 91%, respectively. In contrast, the DT algorithm, with the lowest percentage, only correctly predicted 76% of users with privacy fatigue.

Most of the algorithms had 78% precision. This implies that, among all the users that were predicted to have medium-to-high privacy fatigue, using the SVM, KNN, and RF, 78% truly belonged to this class, and 79% using NB.

**Figure 4:**
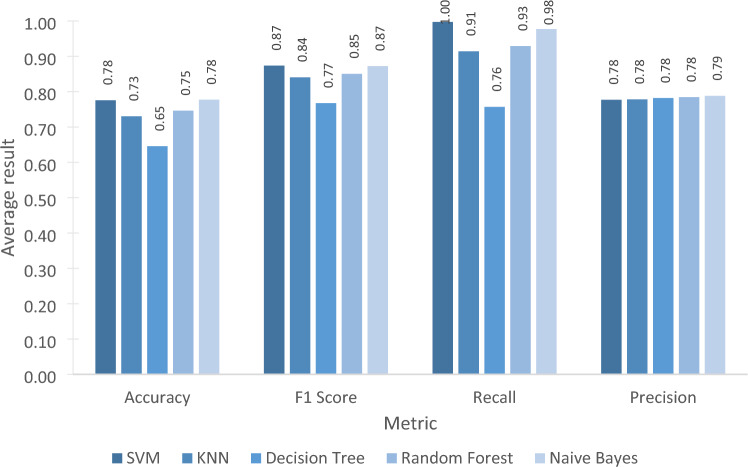
Comparison of models.

In summary, as shown in Fig. 4, the SVM and NB classifiers performed slightly better than the others. Both performed similarly based on the four metrics. This suggests that SVM and NB had greater accuracies in predicting users with privacy fatigue in relation to social media personalized ads based on personality traits and IPA.

## Discussion

The results of this study could be used in several real-world applications. The ML model may help communities to detect users who are more exposed to privacy breaches as a result of privacy fatigue. For example, schools could use this model to find students with a specific personality trait and IPA level to provide courses about online privacy. Based on the results, a student who is careless, impulsive, and aware of information privacy issues is more eligible for this course. They can learn how to protect themselves from privacy breaches and feel less cynical and emotionally exhausted regarding their privacy. On the other hand, the model could also help marketers to promote these kinds of courses to the right audience.

Some weaknesses and strengths of this study compared to previous studies on privacy fatigue (Table 1) should be discussed. A strength of this study is the sample size of 508. 4 out of 5 studies have a lower sample size; only one study has more (620 participants)^17^. Additionally, this study is the only one incorporating ML. Using ML shows how the results could be useful in real life by predicting users likely to suffer from privacy fatigue. On the other hand, most of the related studies used the SEM method^5,6,17,22^, unlike this study. This method ensures the validity and reliability of the measurement model before testing the structural model. This weakness is illustrated next in “Limitations and future work” section.

### Theoretical implications

To the best of our knowledge, there are few studies on the privacy fatigue phenomenon. Therefore, this research provides theoretical support for a better understanding of online privacy behaviors in the social media context. This study also has implications in highlighting that, even if users have a high level of IPA, they will still have privacy fatigue. It also focuses attention on the weak effect of personality traits on privacy fatigue.

Based on the literature, IPA motivates privacy-protective behavior needed for users who suffer from privacy fatigue^30^. In addition, a study^8^ stated that a low level of security and privacy knowledge is likely to increase privacy fatigue, showing a positive relationship. However, our findings showed that IPA negatively correlated with privacy fatigue. This result may corroborate the fact that several studies have stated that individuals’ privacy concerns are associated with their awareness of potential privacy risks^34^. These studies measured awareness by measuring privacy concerns, assuming that a high level of privacy concerns implies a high level of awareness. Therefore, the same could be interpreted with privacy fatigue based on this study's results. The more people are aware of privacy issues, the more frustrated they become.

In various studies, personality traits showed a significant relationship to psychological fatigue^6,7^, contrasting with this study. This could be due to several reasons. One study^6^ concluded that, in different contexts, there is an apparent conflict between the effect of personality traits on privacy fatigue and privacy concerns. The other study^7^, used the long version of BFI (BFI-44) to measure personality traits. In contrast, in this study, the short version (BFI-10) was used here, which could affect the validity and reliability of the results. Therefore, the insignificant effect of personality traits on privacy fatigue could be due to the context of this study or the selected measurements.

### Managerial implications

For managerial implications, social media operators should be aware of the existence and potential effect of privacy fatigue on future services. This psychological state could lead to users’ dissatisfaction with using social media^5^. Policymakers also need to recognize the phenomenon and the extensive use of users’ data. Although governments have set regulations on information privacy, these should be enforced to meet an acceptable level of privacy protection.

### Limitations and future work

This study has some limitations that could be improved in future works. The limitations include the self-report questionnaire, questionnaire validity and reliability, the short version of personality traits’ measurement, and privacy fatigue classification.

The self-report questionnaire is susceptible to response biases because it relies on respondents reporting about themselves^47^. Participants may give answers they consider more socially acceptable, rather than being honest, or may fail to assess themselves accurately. In this study, most participants stated that they are aware of social media practices and their impact, which may not be the case; there is huge uncertainty about social media practices, even among social media owners and developers^48^. Future work may consider collecting data using a different method, such as observation.

The validity and reliability of the study questionnaire were not considered in this study. The SEM method could be used instead of taking the average of the latent variable scores, because it considers the validity and reliability of the questionnaire. SEM was not selected in this study because of the sample size^42^. Additionally, the short version of personality traits was used, with only two questions for each trait. If one of the questions were not valid or reliable, it could not be deleted because SEM calculates validity and reliability based on several questions^49^. Although the validity and reliability of the BFI-10 are affected by factors such as age, culture, and language^39,50^, this study used this scale to capture the overall effect of personality traits on privacy fatigue and not to compare individuals’ differences. However, the long version of personality traits and the SEM method are suggested for use in future research to ensure higher validity and reliability and to capture the full complexity of an individual's personality.

In the ML stage, privacy fatigue was classified into 0 and 1 based on the Likert scale. A study on predicting cyberbullying on social media stated that a comprehensive investigation is required to define and categorize the severity of cyberbullying from social and psychological perceptions^10^. Similarly, efforts from several disciplines are required in the future to identify the levels of severity of privacy fatigue.

Finally, a future study may be conducted based on real privacy-fatigued users. This should involve collaborating with medical professionals/institutes to provide real, comprehensive, and rich samples for more accurate performance.

## Conclusion

This study explored privacy fatigue regarding social media ads. The data were collected using an online questionnaire. The relationships between personality traits and IPA (independent variables) and privacy fatigue (dependent variable) were discovered using regression analysis. ML models were also developed to predict privacy fatigue, using five classification algorithms: SVM, KNN, DT, RF, and NB. The models were evaluated using accuracy, recall, precision, and F1 metrics. The aim was to uncover the impact of social media practices and help target users who may be susceptible to privacy fatigue to motivate their privacy-protective behavior.

The results showed that privacy fatigue exists regarding social media ads. 395 out of 508 participants had a moderate-to-high level of privacy fatigue, which answers the extent of users’ emotional exhaustion and cynicism regarding personalized ads on social media. IPA, i.e., awareness of the collection and use of data for social media ads and its impact had a significant positive relationship with privacy fatigue. Among the five personality traits, i.e., extraversion, neuroticism, openness, conscientiousness, and agreeableness, only the conscientiousness trait had a significant negative association with privacy fatigue. The models with the highest accuracy in predicting privacy fatigue based on IPA and personality traits were SVM and NB, with 78% accuracy and 87% F1.

### Supplementary Information

Supplementary Information 1.

Supplementary Information 2.

## Data Availability

All data generated or analyzed during this study are included in this published article and its Supplementary information files.

## References

[1] Petrosyan, A. *Number of internet and social media users worldwide as of April 2023*. 2023 [cited 2023; Available from: https://www.statista.com/statistics/617136/digital-population-worldwide/.

[2] Epsilon. *New Epsilon research indicates 80% of consumers are more likely to make a purchase when brands offer personalized experiences*. 2018 [cited 2021 1 November, 2021]; Available from: https://www.epsilon.com/us/about-us/pressroom/new-epsilon-research-indicates-80-of-consumers-are-more-likely-to-make-a-purchase-when-brands-offer-personalized-experiences.

[3] Hargittai E, Marwick A (2016). “What can I really do?” Explaining the privacy paradox with online apathy. Int. J. Commun..

[4] Acquisti, A., Friedman, A. & Telang, R. Is there a cost to privacy breaches? An event study. ICIS 2006 proceedings, 94 (2006).

[5] Choi H, Park J, Jung Y (2018). The role of privacy fatigue in online privacy behavior. Comput. Hum. Behav..

[6] Tang, J., Akram, U. & Shi, W. Why people need privacy? The role of privacy fatigue in app users' intention to disclose privacy: Based on personality traits. *J. Enterp. Inf. Manag.* (2020).

[7] Xiao L, Mou J (2019). Social media fatigue-Technological antecedents and the moderating roles of personality traits: The case of WeChat. Comput. Hum. Behav..

[8] Oh J, Lee U, Lee K (2019). Privacy fatigue in the internet of things (IoT) environment. IT CoNverg. PRAct. (INPRA).

[9] Khorrami M, Khorrami M, Farhangi F (2022). Evaluation of tree-based ensemble algorithms for predicting the big five personality traits based on social media photos: Evidence from an Iranian sample. Personal. Ind. Differ..

[10] Al-Garadi MA (2019). Predicting cyberbullying on social media in the big data era using machine learning algorithms: Review of literature and open challenges. IEEE Access.

[11] Albagmi FM (2022). Prediction of generalized anxiety levels during the Covid-19 pandemic: A machine learning-based modeling approach. Inform. Med. Unlocked.

[12] Islam M (2018). Depression detection from social network data using machine learning techniques. Health Inf. Sci. Syst..

[13] Zhu Y-Q, Kanjanamekanant K (2021). No trespassing: Exploring privacy boundaries in personalized advertisement and its effects on ad attitude and purchase intentions on social media. Inf. Manag..

[14] Hayes JL (2021). The influence of consumer-brand relationship on the personalized advertising privacy calculus in social media. J. Interact. Mark..

[15] Lina LF (2021). Privacy concerns in personalized advertising effectiveness on social media. Sriwij. Int. J. Dyn. Econ. Bus..

[16] Pfiffelmann J, Dens N, Soulez S (2020). Personalized advertisements with integration of names and photographs: An eye-tracking experiment. J. Bus. Res..

[17] Agozie DQ, Kaya T (2021). Discerning the effect of privacy information transparency on privacy fatigue in e-government. Gov. Inf. Q..

[18] Hardy G, Shapiro D, Borrill C (1997). Fatigue in the workforce of National Health Service Trusts: Levels of symptomatology and links with minor psychiatric disorder, demographic, occupational and work role factors. J. Psychosom. Res..

[19] Piper, B., Lindsey, A. & Dodd, M. Fatigue mechanisms in cancer patients: developing nursing theory. In *Oncology Nursing Forum*. (1987). 3320981

[20] Mao H (2018). Prevalence and risk factors for fatigue among breast cancer survivors on aromatase inhibitors. Eur. J. Cancer.

[21] Pluut H (2018). Social support at work and at home: Dual-buffering effects in the work-family conflict process. Organ. Behav. Hum. Decis. Process..

[22] Zhu M (2021). Privacy paradox in mHealth applications: An integrated elaboration likelihood model incorporating privacy calculus and privacy fatigue. Telemat. Inform..

[23] Kamalesh MD, Bharathi B (2022). Personality prediction model for social media using machine learning technique. Comput. Electr. Eng..

[24] Sadeghian A, Kaedi M (2021). Happiness recognition from smartphone usage data considering users’ estimated personality traits. Pervasive Mobile Comput..

[25] Joshi ML, Kanoongo N (2022). Depression detection using emotional artificial intelligence and machine learning: A closer review. Mater. Today Proc..

[26] Palos-Sanchez P, Saura JR, Martin-Velicia F (2019). A study of the effects of programmatic advertising on users' concerns about privacy overtime. J. Bus. Res..

[27] Youn S, Shin W (2020). Adolescents’ responses to social media newsfeed advertising: The interplay of persuasion knowledge, benefit-risk assessment, and ad scepticism in explaining information disclosure. Int. J. Advert..

[28] Idberg, L., Orfanidou, S. & Karppinen, O. *Privacy for sale!: An Exploratory Study of Personalization Privacy Paradox in Consumers’ Response to Personalized Advertisements on Social Networking Sites *(2021).

[29] Hillqvist, O. & Johnsson Östergren, A. *The Personalization-Privacy Paradox: Personalized Ads on Social Media: Exploring Invasive Ads on Social Media, in Relation to Perceived Usefulness, Consumer Privacy and Trust*. (2020).

[30] Mamonov S, Benbunan-Fich R (2018). The impact of information security threat awareness on privacy-protective behaviors. Comput. Hum. Behav..

[31] Dhir A (2019). Antecedents and consequences of social media fatigue. Int. J. Inf. Manag..

[32] Clark T (2021). Bryman's Social Research Methods.

[33] Hong, W. & Thong, J. Y. Internet privacy concerns: An integrated conceptualization and four empirical studies*.**Mis Q.* 275–298 (2013).

[34] Correia, J. & Compeau, D. *Information Privacy Awareness (IPA): A Review of the Use, Definition and Measurement of IPA*. In *Proceedings of the 50th Hawaii International Conference on System Sciences,* (2017).

[35] Rammstedt B, John OP (2007). Measuring personality in one minute or less: A 10-item short version of the big five inventory in English and German. J. Res. Person..

[36] Kang, R., *et al*. *“My Data Just Goes Everywhere:” User mental models of the internet and implications for privacy and security*. In *Eleventh Symposium on Usable Privacy and Security (SOUPS 2015)*. (2015).

[37] Harbach, M., Fahl, S. & Smith. M. *Who's afraid of which bad wolf? A survey of IT security risk awareness*. In *2014 IEEE 27th Computer Security Foundations Symposium*. 2014. IEEE.

[38] Gosling SD, Rentfrow PJ, Swann WB (2003). A very brief measure of the Big-Five personality domains. J. Res. Person..

[39] Park J (2022). Comparison of the inter-item correlations of the Big Five Inventory-10 (BFI-10) between Western and non-Western contexts. Personal. Individ. Differ..

[40] Sharma G (2017). Pros and cons of different sampling techniques. Int. J. Appl. Res..

[41] Sriram R (2017). Student Affairs by the Numbers: Quantitative Research and Statistics for Professionals.

[42] Clark, M. *Using Latent Variable Scores*. 2016 December 13, 2022]; Available from: https://m-clark.github.io/docs/lv_sim.html#summary.

[43] Smith TC, Frank E (2016). Introducing machine learning concepts with WEKA. Statistical Genomics.

[44] Scikit Learn. *Cross-validation: evaluating estimator performance*. 2023 [cited 2023 Apr 1, 2023]; Available from: https://scikit-learn.org/stable/modules/cross_validation.html.

[45] Hair JF (2021). A Primer on Partial Least Squares Structural Equation Modeling (PLS-SEM).

[46] Boateng EY, Otoo J, Abaye DA (2020). Basic tenets of classification algorithms K-nearest-neighbor, support vector machine, random forest and neural network: A review. J. Data Anal. Inf. Process..

[47] Kreitchmann RS (2019). Controlling for response biases in self-report scales: Forced-choice versus psychometric modeling of Likert items. Front. Psychol..

[48] Orlowski J (2020). The Social Dilemma.

[49] Sarstedt M, Cheah J-H (2019). Partial Least Squares Structural Equation Modeling Using SmartPLS: A Software Review.

[50] Kunnel John R (2019). Psychometric evaluation of the BFI-10 and the NEO-FFI-3 in Indian adolescents. Front. Psychol..

