# Supplementary material for: A machine learning model to predict privacy fatigued users from social media personalized advertisements
Source: Sci Rep. 2024 Feb 14;14:3685. doi: 10.1038/s41598-024-54078-w (PMC10867113; doi:10.1038/s41598-024-54078-w)
Supplement: Supplementary file 2 — Supplementary Information 2. [file 41598_2024_54078_MOESM2_ESM.html]

Thesis - Classification-CrossValidation


In [1]:

```
# Import required libraries
import numpy as np
import pandas as pd
import matplotlib.pyplot as plt
import seaborn as sns
%matplotlib inline
```

## Data Exploration¶

In [2]:

```
# Read the data
df = pd.read_csv("privacy_fatigue_prediction.csv")
df.head()
```

Out[2]:

|  | extraversion | agreeableness | neuroticism | conscientiousness | openness | IPA | privacy\_fatigue |
| --- | --- | --- | --- | --- | --- | --- | --- |
| 0 | 2.5 | 3.5 | 3.5 | 4.0 | 2.5 | 4.000 | 0 |
| 1 | 4.5 | 3.5 | 2.5 | 4.5 | 3.0 | 3.625 | 0 |
| 2 | 3.0 | 2.5 | 4.0 | 4.5 | 1.5 | 2.000 | 0 |
| 3 | 3.0 | 4.0 | 2.5 | 4.0 | 1.5 | 2.625 | 0 |
| 4 | 2.0 | 5.0 | 3.0 | 4.0 | 4.0 | 4.250 | 0 |

In [3]:

```
# Check null values and data types
df.info()
```

```
<class 'pandas.core.frame.DataFrame'>
RangeIndex: 508 entries, 0 to 507
Data columns (total 7 columns):
 #   Column             Non-Null Count  Dtype  
---  ------             --------------  -----  
 0   extraversion       508 non-null    float64
 1   agreeableness      508 non-null    float64
 2   neuroticism        508 non-null    float64
 3   conscientiousness  508 non-null    float64
 4   openness           508 non-null    float64
 5   IPA                508 non-null    float64
 6   privacy_fatigue    508 non-null    int64  
dtypes: float64(6), int64(1)
memory usage: 27.9 KB
```

In [4]:

```
# Check data statistics
df.describe()
```

Out[4]:

|  | extraversion | agreeableness | neuroticism | conscientiousness | openness | IPA | privacy\_fatigue |
| --- | --- | --- | --- | --- | --- | --- | --- |
| count | 508.000000 | 508.000000 | 508.000000 | 508.000000 | 508.000000 | 508.000000 | 508.000000 |
| mean | 3.438976 | 4.000000 | 2.692913 | 3.805118 | 2.896654 | 3.821604 | 0.777559 |
| std | 0.856128 | 0.637426 | 0.786467 | 0.720517 | 0.798574 | 0.693426 | 0.416296 |
| min | 1.000000 | 2.000000 | 1.000000 | 1.500000 | 1.000000 | 1.000000 | 0.000000 |
| 25% | 3.000000 | 3.500000 | 2.000000 | 3.500000 | 2.500000 | 3.468750 | 1.000000 |
| 50% | 3.500000 | 4.000000 | 2.750000 | 4.000000 | 3.000000 | 3.875000 | 1.000000 |
| 75% | 4.000000 | 4.500000 | 3.000000 | 4.500000 | 3.500000 | 4.250000 | 1.000000 |
| max | 5.000000 | 5.000000 | 5.000000 | 5.000000 | 5.000000 | 5.000000 | 1.000000 |

In [5]:

```
df['privacy_fatigue'].value_counts()
```

Out[5]:

```
1    395
0    113
Name: privacy_fatigue, dtype: int64
```

## Data visualization¶

In [6]:

```
# Visualize Pearson correlation between all variables
plt.figure(figsize=(12,10))
p = sns.heatmap(df.corr(), annot=True, cmap="Blues")
```

## Machine Learning Models¶

In [7]:

```
# Split the data into independent (explanatory) variables and dependent (response) variable
X = df.drop('privacy_fatigue', axis=1)
y = df['privacy_fatigue']
```

In [8]:

```
# Import K-Fold Cross-Validation
from sklearn.model_selection import cross_validate

# Create arrays to store the results
accuracy = []
recall = []
precision = []
f1 = []
accuracy_mean = []
recall_mean = []
precision_mean = []
f1_mean = []

# Method to perform 5-Folds Cross-Validation
# input: the ML classifier, independent variables (X), dependent variable (y), and number of folds
# Output: store the results and the mean of the results of each metric in an array
def cross_validation(model, X, y, cv=5):
    scoring = ['accuracy', 'precision', 'recall', 'f1']
    results = cross_validate(estimator=model,
                           X=X,
                           y=y,
                           cv=cv,
                           scoring=scoring)
    
    # Store metrics of each fold in an array
    # Acuuracy
    accuracy.append(results['test_accuracy'])
    # Recall
    recall.append(results['test_recall'])
    # Precision
    precision.append(results['test_precision'])
    # F1 score
    f1.append(results['test_f1'])  
    
    # Calculate the average of each metric and store it in an array
    # Accuracy mean
    accuracy_mean.append(results['test_accuracy'].mean())
    # Sensitivity (recall) mean
    recall_mean.append(results['test_recall'].mean())
    # Precision mean
    precision_mean.append(results['test_precision'].mean())
    # F1 score mean
    f1_mean.append(results['test_f1'].mean())
```

In [9]:

```
# Import the needed classifiers
from sklearn.svm import SVC
from sklearn.neighbors import KNeighborsClassifier
from sklearn.tree import DecisionTreeClassifier
from sklearn.ensemble import RandomForestClassifier
from sklearn.naive_bayes import GaussianNB

# Create an array of the used classifiers
models = []
models.append(SVC())
models.append(KNeighborsClassifier())
models.append(DecisionTreeClassifier())
models.append(RandomForestClassifier())
models.append(GaussianNB())

# Send all the classifiers to the cross-validation function
for model in models:
    cross_validation(model, X, y, 5)
```

In [10]:

```
# Create an array of models' name
model_list = ['SVM', 'KNN', 'Decision Tree', 'Random Forest', 'Naive Bayes']

# Method to print the metrics for each fold of all the classifiers
# Input: array of the metric result of each fold
# Output: data frame includes the classifier name and the metric result of each fold
def folds_metrics(metric_result):
    # Convert the array to a data frame for better visualization
    metric_df = pd.DataFrame(metric_result, columns = ["1st Fold", "2nd Fold", "3rd Fold", "4th Fold", "5th Fold"])
    
    # add the model name to the data frame
    metric_df.insert(loc=0, column='Model', value=model_list)
    
    # return the result
    return metric_df
```

In [11]:

```
# Print the accuracy for each fold of all the classifiers
folds_metrics(accuracy)
```

Out[11]:

|  | Model | 1st Fold | 2nd Fold | 3rd Fold | 4th Fold | 5th Fold |
| --- | --- | --- | --- | --- | --- | --- |
| 0 | SVM | 0.774510 | 0.774510 | 0.764706 | 0.782178 | 0.782178 |
| 1 | KNN | 0.696078 | 0.715686 | 0.725490 | 0.752475 | 0.762376 |
| 2 | Decision Tree | 0.607843 | 0.656863 | 0.686275 | 0.633663 | 0.643564 |
| 3 | Random Forest | 0.745098 | 0.745098 | 0.725490 | 0.752475 | 0.762376 |
| 4 | Naive Bayes | 0.823529 | 0.784314 | 0.745098 | 0.762376 | 0.772277 |

In [12]:

```
# Print the F1 for each fold of all the classifiers
folds_metrics(f1)
```

Out[12]:

|  | Model | 1st Fold | 2nd Fold | 3rd Fold | 4th Fold | 5th Fold |
| --- | --- | --- | --- | --- | --- | --- |
| 0 | SVM | 0.872928 | 0.872928 | 0.866667 | 0.877778 | 0.877778 |
| 1 | KNN | 0.814371 | 0.832370 | 0.839080 | 0.853801 | 0.862069 |
| 2 | Decision Tree | 0.718310 | 0.774194 | 0.802469 | 0.767296 | 0.775000 |
| 3 | Random Forest | 0.843373 | 0.848837 | 0.839080 | 0.857143 | 0.863636 |
| 4 | Naive Bayes | 0.896552 | 0.875000 | 0.853933 | 0.865169 | 0.871508 |

In [13]:

```
# Print the recall for each fold of all the classifiers
folds_metrics(recall)
```

Out[13]:

|  | Model | 1st Fold | 2nd Fold | 3rd Fold | 4th Fold | 5th Fold |
| --- | --- | --- | --- | --- | --- | --- |
| 0 | SVM | 1.000000 | 1.000000 | 0.987342 | 1.000000 | 1.000000 |
| 1 | KNN | 0.860759 | 0.911392 | 0.924051 | 0.924051 | 0.949367 |
| 2 | Decision Tree | 0.645570 | 0.759494 | 0.822785 | 0.772152 | 0.784810 |
| 3 | Random Forest | 0.886076 | 0.924051 | 0.924051 | 0.949367 | 0.962025 |
| 4 | Naive Bayes | 0.987342 | 0.974684 | 0.962025 | 0.974684 | 0.987342 |

In [14]:

```
# Print the precision for each fold of all the classifiers
folds_metrics(precision)
```

Out[14]:

|  | Model | 1st Fold | 2nd Fold | 3rd Fold | 4th Fold | 5th Fold |
| --- | --- | --- | --- | --- | --- | --- |
| 0 | SVM | 0.774510 | 0.774510 | 0.772277 | 0.782178 | 0.782178 |
| 1 | KNN | 0.772727 | 0.765957 | 0.768421 | 0.793478 | 0.789474 |
| 2 | Decision Tree | 0.809524 | 0.789474 | 0.783133 | 0.762500 | 0.765432 |
| 3 | Random Forest | 0.804598 | 0.784946 | 0.768421 | 0.781250 | 0.783505 |
| 4 | Naive Bayes | 0.821053 | 0.793814 | 0.767677 | 0.777778 | 0.780000 |

In [15]:

```
# Print the mean of the metrics of all classifiers
result = pd.DataFrame({'Model':model_list, 'Accuracy': accuracy_mean, 'F1 Score': f1_mean, 
                       'Recall': recall_mean, 'Precision': precision_mean})
result
```

Out[15]:

|  | Model | Accuracy | F1 Score | Recall | Precision |
| --- | --- | --- | --- | --- | --- |
| 0 | SVM | 0.775616 | 0.873616 | 0.997468 | 0.777131 |
| 1 | KNN | 0.730421 | 0.840338 | 0.913924 | 0.778012 |
| 2 | Decision Tree | 0.645642 | 0.767454 | 0.756962 | 0.782012 |
| 3 | Random Forest | 0.746108 | 0.850414 | 0.929114 | 0.784544 |
| 4 | Naive Bayes | 0.777519 | 0.872432 | 0.977215 | 0.788064 |

In [ ]:

```

```
